# Mask wearing by COVID-19 index cases reduces SARS-CoV-2 transmission to household contacts

**DOI:** 10.1017/S0950268825100642

**Published:** 2025-10-06

**Authors:** Pere Godoy, Jessica Pardos, Manuel García Cenoz, Ignacio Parrón, Iván Martínez-Baz, Joaquim Ferras, Cristina Rius, Sofia Godoy, Diana Toledo, Inma Sanz, Nuria Follia, Carme Miret, Miquel Alsedà, Pedro Plans-Rubió, Monica Carol, Nuria Bes, Maria-Rosa Sala, Joan Caylà, Carmen Muñoz-Almagro, Jesús Castilla, Angela Domínguez, The SARS-CoV-2 Transmission to Household Contacts Working Group (PI21/01883 and ESP22PI01)

**Affiliations:** 1 Institut de Recerca Biomédica (IRB Lleida), Universitat de Lleida, Lleida, Spain; 2 CIBER de Epidemiología y Salud Pública (CIBERESP), Instituto de Salud Carlos III, Madrid, Spain; 3 Agència de Salut Pública de Catalunya, Departament de Salut, Barcelona, Spain; 4 Instituto de Salud Pública de Navarra – IdiSNA, Pamplona, Spain; 5 Servei d’Epidemiologia, Agència de Salut Pública de Barcelona, Barcelona, Spain; 6 Institut Català de la Salut (ICS), Departament de Salut, Lleida, Spain; 7 Facultat de Medicina i Ciències de la Salut, Universitat de Barcelona, Barcelona, Spain; 8 Tuberculosis Research Unit Foundation, Barcelona, Spain; 9 R+D+I Microbiology, Institut de Recerca Sant Joan de Déu, Hospital Sant Joan de Deu, Barcelona, Spain; 10 Medicine Department, Universitat Internacional de Catalunya, Barcelona, Spain

**Keywords:** COVID-19, facemask, household contact, SARS-CoV-2

## Abstract

The objective of this study was to evaluate the impact on SARS-CoV-2 transmission prevention of mask wearing by index cases and their household contacts. A prospective study of SARS-CoV-2 transmission to household contacts aged ≥18 years was conducted between May 2022 and February 2024 in Spain. Contacts underwent a rapid antigen test on day zero and a real-time polymerase chain reaction test 7 days later if results were negative. The dependent variable was SARS-CoV-2 infection in contacts. Index case and contact mask use effects were estimated using the adjusted odds ratio (aOR) and its 95% confidence interval (CI). Studied were 230 household contacts, mean (standard deviation) age 53.3 (16.6) years, and 47.8% (110/230) women. Following index case diagnosis, 36.1% of contacts (83/230) used a mask, and 54.3% (125/230) were exposed to a mask-wearing index case. Infection incidence in contacts was 45.2% (104/230) and was lower in contacts exposed to mask-wearing index cases (36.0% vs. 56.2%; *p* < 0.002). The logistic regression model indicated a protective effect for contacts of both index case mask use (aOR = 0.31; 95% CI: 0.15–0.65) and vaccination (aOR = 0.24; 95% CI: 0.08–0.77). Index case mask use reduced SARS-CoV-2 transmission to contacts, while mask effectiveness was not observed for contacts.

## Introduction

Reducing SARS-CoV-2 transmission in order to prevent COVID-19 cases is a public health priority [1]. Different studies suggest that around 70% of community transmissions occur in homes, and that secondary transmission to contacts of index cases occurs more frequently in households than in any other community setting [2, 3].

The study of household SARS-CoV-2 transmission to contacts is of special interest in the current context, given that most protocols recommend that confirmed COVID-19 cases remain housebound to avoid community transmission [4]. While a number of studies and systematic reviews have examined the factors associated with household transmission, results vary according to geographical area and the viral variant in circulation [5].

Some studies have indicated that index case vaccination, while it does not prevent infection, can reduce transmission to household contacts [6–10]. A history of COVID-19 can also reduce contact susceptibility to infection [11, 12]. Other factors contribute to increased transmission, such as greater exposure due to intimacy or bedroom sharing with the index case, smoking (which damages respiratory system immunity) [13], number of contacts per index case, and non-use of non-pharmacological measures by vaccinated persons due to perceived post-vaccination protection [14].

The highly transmissible nature of the SARS-CoV-2 Omicron variant underscores the critical role of non-pharmacological measures such as face masks [15]. While studies on mask-wearing effectiveness report varying results, masks can be particularly effective in reducing droplet emission by COVID-19 index cases, thereby preventing contact infection [15]. Masks can also be used as a personal protective measure against particles filtered by contacts of index cases, although protection may depend on mask type and its proper use [15].

Clinical trials could provide more precise estimates of the effectiveness of masks in reducing household transmission to contacts, but have the limitation that the effects are studied in highly controlled situations far removed from real-life scenarios. In contrast, observational case–control, cohort, and prevalence studies can yield effectiveness estimates more tailored to specific community transmission situations, but would be subject to different types of biases [15]. Furthermore, mask-wearing effects may differ if studied for COVID-19 index cases (transmission prevention) compared to if studied for contacts (infection prevention) [16, 17].

The aim of this study was to estimate mask use impact on SARS-CoV-2 transmission prevention in index cases and on infection prevention in household contacts, taking into account vaccination and SARS-CoV-2 infection histories of both index cases and contacts.

## Materials and methods

A prospective epidemiological study was conducted, in Catalonia and Navarre (Spain), of COVID-19 index cases and household contacts aged ≥18 years. The methods of the study have been described elsewhere [10], and aspects specific to this study are summarized below.

### Study design

We carried out an epidemiological cohort study of SARS-CoV-2 transmission by index cases to household contacts between May 2022 and February 2024 (when the Omicron variant was circulating) in Spain. COVID-19 cases were identified and selected in eight participating primary care centres at the beginning of each week using rapid antigen testing (RAT) and/or real-time polymerase chain reaction (RT-PCR) testing.

### Participants

Primary care centres were selected, according to convenience criteria, from each epidemiological surveillance area by public health officials attached to the corresponding epidemiological service. Household contacts associated with COVID-19 cases were recruited in a total of eight primary care centres (one in Navarre and seven in Catalonia).

Patient inclusion criteria were as follows: cases positive for SARS-CoV-2 and household contacts ≥18 years who agreed to participate in the study and provided their oral consent (index cases and contacts, respectively). Excluded were individuals with severe and irreversible cognitive, visual disorders, and hearing disorders that hindered their ability to participate in interviews.

Index cases were defined as confirmed cases of SARS-CoV-2 infection in the previous 10 days in a participating centre who had at least one household contact who consented to participate in the study. Household contacts were defined as persons who lived with and were in contact with the index case in the home for at least 2 h in the period from 2 days before the index case diagnosis until inclusion in the study.

### Questionnaire design

A comprehensive literature review was conducted by the coordination committee prior to designing the epidemiological questionnaires [3] in accordance with COVID-19 recommendations of the Spanish Ministry of Health, the World Health Organization, and the European Centres for Disease Prevention and Control. The research team, composed of professionals with epidemiological and public health experience, held several preliminary meetings to decide the different sections, questions, and number of included elements in the questionnaires. Discussions focused on question relevance, consistency, completeness, and clarity, and on questionnaire length.

The definitive questionnaires, obtained after an iterative process of several revisions of previous drafts, included the following sections: sociodemographic data, comorbidities and risk factors, epidemiological information, and knowledge of COVID-19 and preventive measures (face mask use, hand washing, hydroalcoholic solution use, distancing, ventilation, and isolation).

Questions were also included on mask use. Index cases were asked as follows: ‘Did you regularly wear a mask?’ (yes, no, or no reply) and ‘What type of mask did you use?’ (surgical, FP2, or washable). Contacts were asked as follows: ‘After you learned of the index case diagnosis, did you always wear a mask at home?’ (yes, no, or no reply) and ‘What type of mask did you use?’ (surgical, FP2, or washable).

Finally, the questionnaires also included data on previous SARS-CoV-2 infection and COVID-19 vaccination status, validated by checking electronic health records, regional vaccination registers, and epidemiological surveillance databases.

### Data collection

The corresponding questionnaires were administered to the index cases and their household contacts. To detect secondary infections, contacts were followed up for 7 days from confirmation of the index case infection. All contacts underwent RAT on day 0, and those who tested negative underwent RT-PCR testing after 7 days, irrespective of whether or not they were symptomatic.

Data were collected for index cases and contacts as follows: sociodemographic variables (age and sex); date of onset of first symptoms; specific symptoms; diagnostic tests (RAT, RT-PCR); exposure time to the index case; relationship with the index case (cohabitation with a partner, other); shared bedroom with the index case; vaccination history and dates; SARS-CoV-2 infection history and dates; risk factors; and preventive measures following index case diagnosis (mask use, hand washing, hydroalcoholic solution use, distancing, ventilation, and isolation).

Study variable data were collected in an initial face-to-face interview and a subsequent telephone interview. Participants (both index cases and household contacts) who had been vaccinated in the previous 21 days and 7 days were considered vaccinated with a first dose and second dose, respectively. Vaccine effectiveness was studied on the basis of participants having received at least one dose.

### Sampling and sample size

In each participating primary care centre, the first confirmed cases that met the inclusion criteria were selected every 15 days. Subsequently, due to a reduced incidence of new cases, this criterion was expanded to select every week with no limitation on the number. The final sample was composed of 234 household contacts. This sample size, which allowed us to estimate household contact COVID-19 incidence with a precision (e) of ±6% for a 95% confidence interval (CI), was calculated according to the following formulas: *n* = *Z*α^2^ × *p* × (1 − *p*)/*e* ^2^ and *e* = √*Z*α^2^ × *p* × (1 − *p*)/*n.*

### Statistical analysis

The cumulative infection incidence was calculated as the number of infected contacts in the 7 days after symptom onset in the index case (numerator) divided by the number of included contacts (denominator). Index cases were excluded from both the numerator and denominator. The dependent variable was contact SARS-CoV-2 infection (yes/no), and the independent variables were contact exposure to a mask-wearing index case (yes/no) and contact mask use after index case diagnosis (yes/no). The contact covariables were as follows: a previous history of SARS-CoV-2 infection (yes/no); vaccination (yes/no); cohabitation with a partner (yes/no); shared bedroom with the index case (yes/no); exposure to an index case with symptoms; self-isolation; distancing; ventilation; and number of household contacts.

Using a logistic regression model, the adjusted odds ratio (aOR) and the corresponding 95% CI were calculated to determine the association between contact infection (yes/no) and household contact exposure to a mask-wearing index case (yes/no) and household contact mask use after index case diagnosis (yes/no). The variables studied in the multivariate logistic regression model were selected using the backward method for a cut-off point of *p* < 0.2. The variables for household contacts and their interactions evaluated in the model were as follows: exposure to a mask-wearing index case; mask use; age group (years); sex; previous COVID-19; vaccination ≥1 dose; exposure to a vaccinated index case; cohabitation with a partner; shared bedroom; exposure to an index case with symptoms; self-isolation; distancing; ventilation; and number of household contacts.

Household contact exposure to a mask-wearing index case (transmission reduction) and household contact mask use (infection prevention) were calculated, with the corresponding 95% CI, as follows: mask-wearing effectiveness = (1 − aOR) × 100.

Analyses were performed using EpiInfo 7.2.5 (Centers for Disease Control and Prevention, Atlanta, GA) and the SPSS v.24 statistical package (IBM, Armonk, New York, NY).

### Ethical considerations

This study was approved by the Ethics Committee of the Arnau Vilanova University Hospital (code CEIC-2464) and was conducted according to Declaration of Helsinki principles. All subjects included in the study received detailed information on the study aims and granted their consent to participate.

## Results

Of 203 index cases with a mean (standard deviation [SD]) age of 54.6 (19.0) years, 65% (132/203) of whom were women, 98.5% (200/203) had COVID-19 symptoms, 92.6% (188/203) and 88.7% (180/203) had received one and two vaccine doses, respectively, and 45.5% (93/203) regularly wore a mask. Index case mask use was similar for vaccinated patients (54.8% vs. 46.7%; *p* = 0.543), and for patients with comorbidities (52.2% vs. 58.2%; *p* = 0.419), fever (57.2% vs. 47.7%; *p* = 0.202), cough (54.2% vs. 54.1%; *p* = 0.997), or dyspnea (44.0% vs. 55.6%; *p* = 0.274) (Supplementary Table 1).

Of 361 identified contacts, 294 (81.4%) agreed to participate. The study was ultimately conducted with 230 household contacts aged ≥18 years, with a mean (SD) age of 53.3 (16.6) years, 47.8% (110/230) of whom were women; 90.9% were exposed to a vaccinated index case, and 98.3% (226/230) and 90.8% (209/230) were vaccinated with one and two doses, respectively. Over half of the contacts (51.20%, 118/230) had a previous history of COVID-19 (Table 1).

**Table 1.** Incidence of SARS-CoV-2 infection in household contacts of COVID-19 index cases

Variable	Infected contacts n = 104 (%)	Total contacts n = 230
Age (years)		
18–44	27 (41.5)	65
45–64	37 (35.6)	104
≥65	40 (65.6)	61
Sex		
Male	51 (42.5)	120
Female	53 (48.2)	110
Contact vaccination ≥ 1 dose		
Yes	102 (45.1)	226
No	2 (50.0)	4
Contact previous COVID–19 history		
Yes	42 (35.6)	118
No	62 (55.4)	112
Contact mask use		
Yes	39 (47.0)	83
No	65 (44.2)	147
Contact mask type		
None	65 (43.9)	148
FP2	22 (55.0)	40
Surgical	16 (40.0)	40
Washable	1 (50.0)	2
Index case mask use		
Yes	45 (36.0)	125
No	59 (56.2)	105
Index case mask type		
None	59 (56.7)	104
FP2	25 (37.1)	67
Surgical	19 (33.9)	56
Washable	1 (33.3)	3
Index case vaccination		
Yes	89 (42.6)	209
No	15 (71.4)	21
Index case symptoms		
Yes	104 (45.8)	227
No	0 (0.0)	3
Index case self-isolation		
Yes	23 (35.9)	64
No	81 (48.8)	166
Contact distancing		
Yes	63 (49.6)	127
No	41 (39.8)	103
Cohabitation with a partner		
Yes	66 (50.4)	131
No	38 (38.4)	99
Shared bedroom		
Yes	50 (50.0)	100
No	54 (41.5)	130
Contact ventilation		
Yes	103	229 (45.0)
No	1	1 (100.0)
Number of household contacts	1.97*	1.10*

* Mean and standard deviation per case index.

Following index case diagnosis, 36.1% (83/230) of contacts used a mask and 54.3% (125/230) were exposed to a mask-wearing index case. Both index cases and contacts used FP2 masks (53.2% vs. 48.8%) and surgical masks (44.4% vs. 48.8%) in similar proportions, while use of washable masks was negligible (Table 1). Masks were used by 31.3% (72/230) of index cases and contacts and not used by 40.9% (94/230) of index cases and contacts (Table 2).

**Table 2.** Face mask use by COVID-19 index cases and household contacts

	Index case masking		
Contact masking	No (%)	Yes (%)	Total
No	94 (63.95)	53 (36.05)	147
Yes	11 (13.25)	72 (86.75)	83
Total	105 (45.65)	125 (54.35)	230

Odds ratio = 11.6; 95% confidence interval: 5.6–23.8; *p* < 0.001.

Infection incidence among contacts was 45.2% (104/230) and was similar in men and women (42.5% vs. 48.2%; *p* = 0.387). It also differed relatively little for vaccinated contacts (45.1% vs. 50.0%; *p* = 0.846), self-isolation (35.9% vs. 48.8%; *p* = 0.079), distancing (39.8% vs. 49.6%; *p* = 0.137), exposure to an index case with symptoms (*p* = 0.285), ventilation (*p* = 0.270), number of household contacts (*p* = 0.451), and mask wearing (47.0% vs. 44.2%; *p* = 0.685) (Table 3). Incidence increased with age, was higher in persons aged ≥65 years compared to persons aged 18–29 years (65.6% vs. 41.5%; *p* < 0.001), in contact partners of index cases (50.4% vs. 38.4%; *p* < 0.046) and in persons with no previous history of COVID-19 (55.4% vs. 35.6%; *p* < 0.001). Incidence was lower in contacts exposed to mask-wearing index cases (36.0% vs. 56.2%; *p* < 0.002) and to vaccinated index cases (42.6% vs. 71.4%; *p* < 0.011) (Table 3).

**Table 3.** Factors associated with SARS-CoV-2 infection in household contacts of COVID-19 index cases

Variable	Contacts				*p* value
	Infected *n* = 104	Non-infected *n* = 126	OR	95% CI	
Age ± SD	56.6 ± 17.0	50.6 ± 15.7			0.006
Age (years)					
18–44	27	38	1.00		
45–64	37	67	0.78	0.41–1.48	0.437
≥65	40	21	2.66	1.29–5.56	<0.007
Sex					
Male	51	57	0.79	0.47–1.34	0.387
Female	53	69	1.00		
Contact vaccination ≥ 1 dose					
Yes	102	124	0.82	0.11–5.94	0. 846
No	2	2	1.00		
Contact mask type					
None	65	83	1.00		
FP2	22	18	1.56	0.77–3.18	0.212
Surgical	16	24	0.85	0.41–1.74	0.657
Washable	1	1	1.23	0.03–50.37	0.999
Contact previous COVID–19 history					
Yes	42	76	0.44	0.26–0.76	0.003
No	62	50	1.00		
Contact mask use					
Yes	39	44	1.11	0.65–1.92	0.685
No	65	82	1.00		
Index case mask use					
Yes	45	80	0.44	0.25–0.74	0.002
No	59	46			
Index case mask type					
None	59	45	1.00		
FP2	25	42	0.46	0.24–0.85	0.013
Surgical	19	37	0.39	0.20–0.77	0.006
Washable	1	2	0.38	0.01–5.19	0.421
Index case vaccination					
Yes	89	120	0.30	0.11–0.79	0.011
No	15	6	1.00		
Index case symptoms					
Yes	104	123	–	–	0.285
No	0	3	1.00		
Index case self-isolation					
Yes	23	41	0.60	0.33–1.08	0.043
No	81	86	1.00		
Contact distancing					
Yes	41	62	1.00	–	
No	63	64	1.49	0.88–2.52	0.137
Cohabitation with a partner					
Yes	66	65	1.63	0.96–2.78	0. 046
No	38	61	1.00	–	
Shared bedroom					
Yes	50	50	1.41	0.83–2.38	0.201
No	54	76	1.00		
Contact ventilation					
Yes	103	126	–	–	0.270
No	1	0	1.00		
Number of household contacts, mean ± SD					
	1.91 ± 1.03	2.02 ± 1.15			0.451

CI, confidence interval; OR, odds ratio; SD, standard deviation.

In the logistic regression model, being a partner of index cases was associated with a higher risk of infection (aOR = 2.81; 95% CI: 1.20–6.58), while a history of previous COVID-19 was a protective factor against new infections (aOR = 0.35; 95% CI: 0.18–0.68). Index case mask use (aOR = 0.31; 95% CI: 0.15–0.65) and index case vaccination (aOR = 0.24; 95% CI: 0.08–0.77) both showed a protective effect in preventing contact infection (Table 4).

**Table 4.** Multivariate logistic regression of factors associated with household SARS-CoV-2 transmission

Variable	aOR	95% CI	*p* value
Age (years)			
18–44	1.00		
45–64	0.60	0.28–1.24	0.169
≥65	1.56	0.65–3.76	0.321
Sex			
Male	0.68	0.36–1.27	0.223
Female	1.00		
Contact vaccination ≥1 dose			
Yes	1.20	0.12–11.93	0.871
No	1.00		
Contact previous COVID–19 history			
Yes	0.35	0.18–0.68	0.002
No	1.00		
Contact mask use			
Yes	2.03	0.95–4.35	0.065
No	1.00		
Index case mask use			
Yes	0.31	0.15–0.65	0.002
No	1.00		
Index case vaccination			
Yes	0.24	0.08–0.77	0.017
No	1.00		
Index case self-isolation			
Yes	0.72	0.34–1.52	0.388
No	1.00		
Cohabitation with a partner			
Yes	2.81	1.20–6.58	0.017
No	1.00		
Shared bedroom			
Yes	1.02	0.49–2.11	0.967
No	1.00		
Number of household contacts	1.13	0.84–1.51	0.412

aOR, adjusted odds ratio (according to the remaining variables in the table); CI, confidence interval.

## Discussion

The study found that, in a high household SARS-CoV-2 transmission scenario, index case mask use reduced the risk of contact infection. Mask use by contacts was strongly associated with mask use by index cases, but no protective effect was observed, probably due to use after index case diagnosis when transmission may have already occurred. The study also found that index case vaccination reduced the risk of transmission to contacts and that a history of COVID-19 in contacts reduced their susceptibility to further infection.

Mask use by index cases reduces infection transmission to contacts by avoiding or reducing the dissemination of infected aerosols [16, 17]. The effectiveness observed in our study (69%) falls in the high range of rates observed in other studies [15] and in the meta-analysis by Brainard et al. [18]. In a retrospective study of 335 people in 124 families, Wang et al. [19] reported a similar impact of mask use on transmission, estimating 79% effectiveness when index cases and their contacts used masks [19]. A very similar effectiveness of 70% was also reported for an observational study of 382 sailors on the USS Theodore Roosevelt aircraft carrier [20]. Other studies of viral load in exhaled breath [21, 22] have reported that people with mild or asymptomatic SARS-CoV-2 infection shed infectious aerosols, that mask use significantly controlled the source of infection, and that masks reduced viral aerosols in indoor air by up to half. These results are consistent with those of a recent study that found a reduced viral load in the breath of masked COVID-19 patients (any type of mask), leading to a significant reduction in transmission [16].

Mask use by contacts, which aims to reduce susceptibility to infection by filtering out virus-containing aerosols, was strongly associated with mask use in index cases (*p* < 0.001); however, no protective effect in reducing infection was observed in the logistic regression analysis. Note that contacts only started wearing masks after learning of index case diagnosis, but by then, they may have already been infected, especially if the index case was not wearing a mask. Other studies also point to lower mask effectiveness in persons exposed to filtered aerosols, especially when mask use begins after index case diagnosis [18]; Wang et al. [19], for instance, reported no protective effect for household contacts if masks were used after learning of index case diagnosis. Likewise, Elgersma et al., in a cross-sectional study of 3,209 participants, found no effectiveness in reducing the risk of SARS-CoV-2 infection while wearing masks outdoors most of the time [23]. This results contradict previous studies, both experimental and observational, on the effectiveness of mask use on the risk of infection [24, 25], which reported that mask use reduced the risk of SARS_CoV-2 infection. An experimental study further confirms that the protection provided by masks is greater when the mask is worn by the index case [26]. In a Californian test-negative case–control study, mask effectiveness was estimated at 66% if worn for 2 weeks prior to study entry [27].

Similar to what happens with the group protection provided by vaccination, the more widely an intervention is adopted by a community (in this case, mask use by COVID-19 cases), the greater the benefit for all its members. Mask use prevalence might be even more important than the type of mask worn [15]. In our study, household mask use as reported by COVID-19 index cases was 54.3% – a percentage that could be raised by recommendations from primary care physicians. While mask wearing is acknowledged to both be uncomfortable (especially over prolonged periods in hot environments) and to inhibit verbal and non-verbal communication, mask use at home by an index case would be limited in time and would lead to a significant reduction in transmission of infection to contacts. Regarding oxygen saturation reduction and carbon dioxide retention concerns on using a mask, these have not been confirmed in available studies [28]. It is not unreasonable to suppose that persons with more severe symptoms might be less likely to wear a mask (due to discomfort, breathing difficulties, coryza, or cough) and so would be more likely to transmit infection onwards. However, although almost all of the index cases in our study (98.5%; 200/203) were symptomatic, we observed no statistical differences in mask wearers with fever, cough, or dyspnea (Supplementary Table 1). Note, however, that the study might not have been sufficiently powered to explore this possibility.

A notable finding was the effectiveness of index case vaccination in reducing transmission to contacts. In a previous study, we documented the effectiveness of 79% (95% CI: 93%–33%) [10], and we report a similar high effectiveness of 76% (95% CI: 92%–33%) in this study, once the effect of index case masking and contact infection history was taken into account. Similar results have been observed in other studies that, using different methodologies, have estimated reductions of 40% to 80% in household transmission of infection [8, 9, 29]. These results, which highlight the important role of vaccination in reducing SARS-CoV-2 transmission, support vaccination policies for people in contact with vulnerable persons in their homes and for vulnerable workers, for example, nursing home and healthcare employees.

Previous SARS-CoV-2 infection provides important, albeit relative, protection. The 65% effectiveness observed in this study – broadly similar to the 56% and 51% observed by Altarawaneh et al. [11] and Suarez-Castillo et al. [12], respectively – confirms immune protection from previous infection that can also be enhanced by booster doses [30, 31, 32]. Previous infection is a variable that should be taken into account in estimating both index case and contact vaccination effectiveness and the effectiveness of non-pharmacological measures.

Some studies report lower transmission as the number of household contacts increases [33, 34]. While Bernal et al. [33] documented this effect, it was only statistically significant for households with five or more contacts. This effect has been associated with the higher likelihood of transmission in smaller households where the main contact is the index case’s partner. In our study, the index case’s partner had triple the risk of infection.

This study has certain limitations. Data on mask use were not collected through direct observation, and for the index cases, biased responses regarding mask use may be affected by perceptions of social acceptability. The information about mask use by contacts was recorded before clinical samples were collected and so was not influenced by the outcome of a possible infection. Simply asking cases, ‘Did you regularly wear a mask?’ is likely a non-specific question, and it could be argued that it would have been better to ask about the circumstances under which a mask was worn in the home; however, this information was not available in this study. There is also the possibility that mask wearers feel somewhat protected and thus change their behaviors to not observe social distancing, so that any benefit of masking is offset by increased exposure. In any case, no protective effect was observed in mask use by contacts, probably due to use after index case diagnosis when transmission may have already occurred. Due to the small number of participants, the type of mask used was not analyzed in the logistic regression model; however, Lai et al. [16] observed a reduction in viral load for all mask types. Infections in vaccinated individuals may have been underestimated, due to fewer or subclinical symptoms, although all subclinical cases in our study were likely to have been detected, as contacts underwent RAT and those who tested negative underwent RT-PCR 7 days later regardless of symptoms. Self-isolation of COVID-19 cases, social distancing, and ventilation can reduce transmission, although these measures are generally difficult to implement in most households. In our study, their effectiveness could not be directly observed and it is difficult to determine this kind of effectiveness through interviews. Some studies indicate lower transmission as the number of household contacts increases. Although this variable and being the index case’s partner were included in the logistic regression model, we cannot rule out residual confounding.

A strength of the study is that regular home use of masks was prospectively recorded and that contacts were classified as infected or non-infected based on laboratory results. Furthermore, information on contact exposure was collected before laboratory results were known, and all information on vaccination and COVID-19 history was validated by checking clinical records and epidemiology databases.

Our main conclusion is that index case mask use reduces SARS-CoV-2 transmission to contacts. Mask use by contacts was not observed to be effective, possibly due to delayed use after the index case diagnosis was known. Index case vaccination also reduces SARS-CoV-2 transmission to contacts. The finding that COVID-19 index case mask use and vaccination both play a key role in reducing household transmission suggests that public health services, and especially primary care centres, need to focus on ensuring household mask use when diagnosed COVID-19 cases are instructed to remain housebound. To date, most mask recommendations have been based on observational studies with limited evidence. More randomized trials or quasi-experimental studies are needed to better understand the effectiveness of masks in protecting against the transmission of respiratory pathogens.

## Supporting information

10.1017/S0950268825100642.sm001 Godoy et al. supplementary material Godoy et al. supplementary material

## Data Availability

The data presented in this study are available on request from the corresponding author.

## Supplementary material

The supplementary material for this article can be found at http://doi.org/10.1017/S0950268825100642.

## The SARS-CoV-2 Transmission to Household Contacts Working Group (PI21/01883 and ESP22PI01) members

Pere Godoy (PG), Manuel García-Cenoz (MGC), Miquel Alsedà (MA), Gloria Carmona (GC), Pere Plans (PP), Pilar Ciruela (PC), Nuria Follia (NF), Nuria Bes (NB), Núria Soriano (NS), Ignasi Parrón (IP), Caritat Planas (CP), Irene Barrabeig (IB), Mònica Carol (MC), Joaquim Ferras (JF), Montserrat Guillaumes (MG), David Palma (DP), Diana Toledo (DT), Iván Martínez-Baz (IMB), Carmen Muñoz-Almagro (CMA), Cristina Rius (CR), Glòria Perez (GP), Maria-Rosa Sala (MRS), Joan A Caylà (JAC), Ángela Domínguez (AD), Jesús Castilla (JC), Sofia Godoy (SG), Jessica Pardos (JP), Carme Miret (CM), Mariona Vilar Pont (MVP), Aroa Illa Casarramona (AIC), Joaquim Solà Pou (JSP), Ivett Morales Arteaga (IMA), Blanca Manuel Marti (BMM), Javier Remón Piñol (JRP), Inmaculada Sanz (IS), Jose Abadin Barrantes (JAB), Alex Ortega Roca (AOR), Pablo Aldaz (PA), Cristina Burgui (CB), Alexandre Ortega Roca (AOR), Raquel Hurtado Portero (RHP), Victor Guadalupe (VG), Anna Vilalta (AV), Montserrat Zayas (MZ).
