# Supplementary material for: Mask wearing by COVID-19 index cases reduces SARS-CoV-2 transmission to household contacts
Source: Epidemiol Infect. 2025 Oct 6;153:e125. doi: 10.1017/S0950268825100642 (PMC12571686; doi:10.1017/S0950268825100642)
Supplement: Godoy et al. supplementary material [file S0950268825100642sup001.docx]

**Supplementary table 1.** Characteristics of index cases of COVID-19 wearing masks

| **Variable** | **cases of COVID-19 wearing masks** | | **OR*** | **95% CI*** | **P value** |
| --- | --- | --- | --- | --- | --- |
|  | **Yes**  **n=110** | **No**  **n=93** |  |  |  |
| Age ± SD* | 54.5 ± 21.6 | 54.6 ± 16.6 |  |  | 0.961 |
| **Sex** | | | | | |
| Male | 35 (49.3) | 36 (50.7) | 1.35 | 0.76-2.41 | 0.305 |
| Female | 75 (56.8) | 57 (43.2) | 1.00 |  |  |
| **Risk factors (comorbidities)** | | | | | |
| Yes | 71 (52.2) | 65 (47.8) | 0.78 | 0.43-1.42 | 0.419 |
| No | 39 (58.2) | 28 (41.8) | 1.00 |  |  |
| **Index case previous COVID-19 history** | | | | | |
| Yes | 49 (59.4) | 34 (41.0) | 1.39 | 0.79-2.45 | 0.248 |
| No | 61 50.8) | 59 (49.2) | 1.00 |  |  |
| **Index case vaccination** | | | | | |
| Yes | 103 (54.8) | 85 (45.2) | 0.72 | 0.25-2.07 | 0.543 |
| No | 7 (46.7) | 8 (53.3) | 1.00 |  |  |
| **Index case symptoms** | | | | | |
| Yes | 109 (54.5) | 91 (45.5) | 2.39 | 0.18-71.27 | 0.874 |
| No | 1 (33.3) | 2 (66.7) | 1.00 |  |  |
| **Fever** | | | | | |
| Yes | 79 (57.2) | 59 (42.7) | 1.47 | 0.81-2.65 | 0.202 |
| No | 31 (47.7) | 34 (52.3) | 1.00 |  |  |
| **Cough** | | | | | |
| Yes | 84 54.2) | 71 (45.8) | 1.00 | 0.52-1.92 | 0.997 |
| No | 26 (54.2) | 22 (45.8) | 1.00 |  |  |
| **Dyspnoea** | | | | | |
| Yes | 11 (44.0) | 14 (56.0) | 0.63 | 0.27-1.46 | 0. 275 |
| No | 99 (55.6) | 79 (44.4) | 1.00 | - |  |

*CI: confidence interval; OR: odds ratio; SD: standard deviation.
